# The HOPE4MCI study: AGB101 treatment reduces entorhinal cortex atrophy in patients with amnestic mild cognitive impairment due to Alzheimer’s Disease who are ApoE‐4 non‐carriers

**DOI:** 10.1002/alz.092376

**Published:** 2025-01-09

**Authors:** Nisha Rani, Richard Mohs, Michela Gallagher, Arnold Bakker

**Affiliations:** ^1^ Department of Psychiatry and Behavioral Science, Johns Hopkins University School of Medicine, Baltimore, MD USA; ^2^ AgeneBio, Inc., Baltimore, MD USA; ^3^ Department of Psychological and Brain Sciences, Johns Hopkins University, Baltimore, MD USA; ^4^ Department of Neurology, Johns Hopkins University School of Medicine, Baltimore, MD USA

## Abstract

**Background:**

Hippocampal hyperactivity is a hallmark of prodromal Alzheimer’s disease (AD) that predicts progression in patients with amnestic mild cognitive impairment (aMCI). AGB101 is an extended‐release formulation of levetiracetam in the dose range previously demonstrated to normalize hippocampal activity and improve cognitive performance in aMCI. The HOPE4MCI clinical trial used a 78‐week protocol to assess progression in amyloid‐positive patients with MCI due to AD. Mohs et al. (2024) reported the clinical/cognitive results showing that ApoE‐4 non‐carriers had a more favorable treatment effect of AGB101 over the 78‐week duration of the study. Here we report effects of AGB101 on biomarkers of AD in those patients.

**Methods:**

Forty‐four participants who completed the 78‐week protocol were ApoE‐4 non‐carriers. Structural MRI scans obtained in the study at baseline and after 78 weeks were analyzed using the Automated Segmentation of Hippocampal Subfields (ASHS) software providing volume measures of key structures of the medial temporal lobe relevant to AD progression. Blood samples collected at 78 weeks in the study were also analyzed for plasma biomarkers in those participants.

**Results:**

Treatment with AGB101 significantly reduced atrophy in the left entorhinal cortex (p < 0.05) compared to placebo. Reduced atrophy in the entorhinal cortex was significantly correlated with the change in CDR‐SB score (r = ‐0.511) over 78 weeks, as well as with biomarkers of neurodegeneration neurofilament light (NfL; r = ‐0.382) and glial fibrillary acidic protein (GFAP; r = ‐0.398) collected at completion of the protocol.

**Conclusion:**

As reported in Mohs et al. (2024), progression on the primary clinical/cognitive endpoint of the Clincial Dementia Rating Scale Sum of Boxes score (CDR‐SB) occurred over the 78‐week protocol in the HOPE4MCI study and non‐carriers of ApoE4 treated with AGB101 showed a substantially more favorable effect than carriers. Here we report that treatment with AGB101, in those participants significantly reduced atrophy of the entorhinal cortex. That reduction in atrophy was significantly coupled with the change in CDR‐SB and with plasma biomarkers of disease. These exploratory analyses would be consistent with a reduction in neurodegeneration in the non‐carriers of ApoE4 treated with AGB101 prior to a clinical diagnosis of dementia.

